# Assessing the need and capacity for integration of Family Planning and HIV counseling and testing in Tanzania

**Published:** 2012-12-25

**Authors:** Bayoum Awadhi, Beati Mboya, Florence Temu, Zubeda Ngware

**Affiliations:** 1 African Medical and Research Foundation (AMREF), Dar es Salaam, Tanzania; 2 Tanzania Field Epidemiology and Laboratory Training Program (FILTEP), Dar es Salaam, Tanzania

**Keywords:** Family planning, HIV Counseling and testing, Integration, service

## Abstract

**Introduction:**

In Tanzania, only 27% of currently married women are using modern Family Planning (FP) methods. HIV counseling and testing outlets are potential avenues for addressing the unmet FP needs through integration of FP and HIV services, reaching out to sexually active people. This study assessed capacity for integrating family planning into HIV voluntary counseling and testing services (HTC) in Dar-es-Salaam (DSM) and Coast regions.

**Methods:**

This was a mixed methods study using triangulation model conducted in two districts to represent rural and urban settings. Questionnaires were administered to 147 randomly selected service users and 35 health providers while 10 in-depth interviews were conducted among Ministry of Health and Local government. Four focus group discussions were conducted among HIV voluntary counselling and testing (VCT) service users. Quantitative data was analyzed using descriptive statistics with aid of SPSS. Qualitative data were analyzed by thematic framework approach.

**Results:**

Although there was gap in policy and guidelines with regards to integration, policy makers were willing to pioneer integration of FP and HIV services. Health providers support provision of FP/VCT services by the same health provider. Only 25% of health providers were trained on both FP and HTC services. Existing national monitoring and evaluation tools can be used with little modification to capture data for both services. Eighty five percent (85%) of clients indicated satisfaction with integrated services.

**Conclusion:**

Integration of FP and HTC is feasible and acceptable with minor re-arrangement. Involvement of multiple stakeholders especially at district level is critical in enhancing integration.

## Introduction

Although the rate of new HIV infection has decreased, UNAIDS reports show that the number of people living with HIV in Sub Saharan Africa continues to rise and in 2009 the number reached 22.5 million contributing to 68% of total global burden of the disease. Sexually active youth comprise 60% of all HIV infections in Sub Saharan Africa [1]. In Tanzania HIV prevalence has decreased from 7.0% in 2004 to 5.7% in 2008 among men and women aged 15-49 years [2, 3]. Around 1.2 million people aged 15-49 years, or just over 5 percent of the adult population, are living with HIV in Tanzania [3]. Majority of people in Tanzania acquire HIV through sexual intercourse (80%) and the population most severely affected are sexually active youth (75%) particularly women [1]. It is therefore important that HIV service provision incorporates the sexual and reproductive needs of HIV infected youth, particularly women, family planning inclusive.

Although contraception offers a variety of benefits for the mother, her family, and the community at large, unmet need for FP averages 19.4% in Sub-Saharan Africa [4].In Tanzania despite a high fertility rate (5.7%) there is a high unmet family planning need; 22% among married women aged 15-49 years and 23% among young women aged 20-24 [2, 3]. The unmet need is higher among HIV positive women as evidenced by low utilization of FP services (26%) among HIV positive women attending postnatal services against a national target of 80% [3]. According to the 2010 Tanzania Demeographic Health Survey, condom use that provides double protection against HIV and conception was as low as 27% of women and 23.6% of men; younger respondents are more likely to use condoms than older respondents [5].

The potential benefits of integrating these services are increasingly apparent and the WHO, UNAIDS and several global health bodies have provided guidance on this [6–9]. The Government of Tanzania has committed to promote, facilitate and support in an integrated manner, the provision of FP services in Tanzania [10]. Coverage of family planning services in Tanzania is still low where approximately three-fourths (76%) of Tanzanian health facilities offer some temporary modern methods of family planning [5]. Although contraceptive prevalence has doubled from 10% in 1992 to 22% in 1999, access to FP particularly for youth, and meeting their needs continues to be a challenge. This is evidenced by frequent occurrences of unplanned pregnancies, unwanted births and unsafe abortions among adolescents and young women; the people who are at most risk of HIV infection. Many HIV-infected women likely need family planning services, but unmet need for these services is often greatest in countries with high HIV prevalence. This need can be better met if family planning services are offered where such women access HIV related services, in addition to being offered through family planning programs.

VCT sites are potential avenues to explore integration of FP and HIV services as both services aim at reaching sexually active people and promoting safe and healthy sexuality, including the prevention of HIV, STIs, and unwanted pregnancy regardless of one's HIV sero status. However at VCT sites, there are obvious missed opportunities to provide FP services, as often times clients express the need but do not access the services. For instance in a 2002 assessment of VCT centers in Tanzania it was found that over one-half of sexually active VCT clients reported that they did not use contraceptive methods [16]. The client-provider interaction during VCT sessions can provide an opportunity to incorporate FP information, counseling and provision of methods. However, this service is not offered in VCT settings. This is a missed opportunity that needs to be exploited to address the unmet need for contraception for VCT clients.

Evidence from operational research conducted by Family Health International (FHI) in Kenya, demonstrated feasibility and cost effectiveness of FP/VCT integration and high acceptability of clients to receive contraception in VCT settings. Nearly 8% of women who visited VCT centers were estimated to be HIV infected. Many VCT clients were interested in avoiding pregnancy but were not currently using FP methods. This result had important implications for enforcing integration into other settings [11]. In Uganda, a high demand for FP was observed upon increasing the VCT clients’ knowledge for FP services [12]. Studies that have explored integrated FP and VCT services and the outcome in the pilot phase found that health providers are supportive of service integration and that challenges exist but may be overcome with supportive supervision and adequate training [13].

Despite the fact that that family planning and HIV prevention are complimentary to each other, in most settings FP and HIV/AIDS services have traditionally been offered separately. Evidence on the most effective practical approaches and models of FP/VCT integration which could be adopted at different levels of health facilities in low resource settings particularly in Tanzania is required. AMREF through ANGAZA VCT Project has pioneered the implementation of the government VCT policy to ensure accessibility and availability of VCT services through supporting establishment of VCT sites in rural and urban settings. In 2008 AMREF through ANGAZA VCT project conducted this study to assess capacity for integrating family planning into HIV voluntary counseling and testing services in Dar es Salaam (DSM) and Coast regions. The purpose of the study was to assess the capacity to implement integrated FP/VCT services in DSM and Coast Regions.

Along with the main objective the study assessed existing policies, guidelines, systems and structures at different levels with regards to integrated FP/VCT programming. It also assessed the health care provider and clients’ perception towards FP/VCT integration. Family planning utilization and service gap among clients visiting HIV counseling and testing outlets was also determined.

## Methods

This mixed methods study using triangulation model was conducted in two districts in Dar-es-Salaam and two districts in Coast region, Tanzania [14]. It involved concurrent collection of data, and integrates both quantitative and qualitative data in the results, interpretation and conclusion. The districts surveyed included Ilala, Kinondoni, Kisarawe and Bagamoyo including the following sites; AMREF Training Center, Mnazi Mmoja, Magomeni, AFIKI and Bagamoyo district hospital. The study populations comprised of Ministry of Health Officials, in-charges of health facilities, service providers, Family Planning and Voluntary Counseling and Testing service users. Five facilities were selected purposively for the FP/VCT piloting using the following selection criteria: representation of urban and rural set up, representation of various types of service delivery outlets i.e. health centre, stand alone and mobile, ownership i.e. public and private and willingness to participate. Purposive sampling was employed for the Ministry of Health Officials, in-charges of health facilities participated in semi-structured and key informant interviews. All counselors working in counseling and testing sections and health providers working in family planning and sexual reproductive health in each selected health facility were interviewed. Random sampling was used for FP and VCT service users who participated in questionnaire survey. In total 4 focus group discussions were conducted, 10 policy makers were interviewed, while questionnaire was administered to 35 health care providers and 147 service users.

Mixed methods were used to collect different views including semi structured interviews, key informant interviews, structured questionnaire, and focus group discussions. A pre-survey training session was conducted for all interviewers who participated. Pretesting, revision and adjustment of the data collection instruments was done before the actual data collection process. Information collected included trainings, skills, knowledge, attitudes and experience in family planning methods and voluntary counseling and testing.

The quantitative data were entered onto a computer and cleaned using EPI Info and thereafter analyzed by Pearson Chi-square (X2) using Statistical Package for Social Sciences (SPSS) version 16. All qualitative data were recorded in a radio-cassette and transcribed immediately after each interview and FGD and stored in electronic form as text as well as in audio form. MAXQDA software package was used to help manage the qualitative data during the analysis especially in organizing and retrieving data. Five steps of the framework approach for qualitative data analysis were followed to analyze the textual data. A thematic framework was drawn from the research objectives and issues arose from the interviews. The coding system used during analysis was developed and adjusted by the research team based on research objectives. The data were indexed and grouped into themes and subthemes according to level of generality for easy retrieval, review and further exploration. Then all related segments from different transcripts were pulled together and examined to identify concepts and provide explanation for the findings. Triangulation of findings through use of focus group discussions, key informant interviews and questionnaires was done in order to improve comprehensiveness of data collected and increase credibility of the findings. In order to enhance data accuracy, site supervisors edited the quantitative data each evening; errors were corrected and the questionnaires were packed in safe envelopes and transported to AMREF offices, Dar-es-Salaam.

Ethical clearance was sought from National Institute of Medical Research. Consent to participate in the VCT and FP interviews were obtained according to the current national guidelines. In this study, confidentiality and privacy of the respondents was ensured by taking steps like using numbers instead of names to maintain anonymity during the collection, storage and entry of the data.

## Results

A total of 147 services users participated in this study through completing standardized questionnaires. Majority were females (79.6%), a large proportion had primary school education (61.9%). Table 1 summarizes various social demographic characteristics of the participants. All 35 health care providers from the 5 study sites (Table 2) were involved in the study, 88.6% of them being females. More than half of the respondents had more than 5 years work experience. Majority of the respondents (60%) were nurses.

**Table 1:** Socio demographic characteristics of services users

Socio-demographic characteristics	Frequency	Percent
**Sex**		
Male	30	20.4
Female	117	79.6
**Age**		
15-24	47	32.0
25-34	70	47.6
35+	30	20.4
**Occupation**		
Business	42	28.6
Employed	56	38.1
Not Employed	49	33.3
**Education**		
Primary	91	61.9
Secondary	36	24.5
Above Secondary	20	13.6
**Marital Status**		
Single	61	41.5
Married	70	47.6
Others	16	10.9

**Table 2:** Socio-demographic characteristics of health providers

Socio-demographic characteristics	Frequency	Percent
**Sex**		
Male	4	11.4
Female	31	88.6
**Years worked (experience)**		
≤ 5 years	16	45.7
Above 5 Years	19	54.3
**Job Title**		
Nurse	21	60.0
Nurse Assistant	1	2.9
Others	13	37.1

### Policies and guidelines influencing integrated FP/VCT programming

Majority of health providers interviewed (71.4%) said there are no guidelines or protocols for integrated services. Commonly, policy makers and service providers reported that there are no current strategies addressing integration. The government through the ministry of health is currently developing a strategy for advocating integration of family planning and HIV/AIDS services.

*“Currently there is no strategy addressing integration. However the government through MOHSW is planning to develop a strategy to advocate integration of FP/ HIV services”* Male Health provider.

Specifically, Reproductive and Child Health (RCH) Policy Guidelines, Health Policy, HIV Policy, HIV Treatment Guidelines were mentioned as examples of guidelines that support condom use. Policy makers commonly mentioned protocols that are specifically geared towards integration of family planning and HIV services include Family planning, HIV care and treatment Curriculum 2009, Service providers’ decision making toolkit, PMTCT guideline, HBC guideline, RCH guideline, STI guidelines, and CTC guidelines.

*“Yes, the national FP strategy/policy includes HIV prevention, treatment, care and support issues. These policies include HSSP II, STI Guidelines, PMTCT Guidelines, and RCH Guidelines”* MOHSW Reproductive health Officer.

Integration of FP and HTC services was perceived as a way forward for improving access to services especially where health providers are scarce compared to number of clients. Involvement of various stakeholders in design and implementation of integrated services was echoed by decision makers.

*“All level of decision makers (National and District) need to be well informed and sensitized on the integration”* District Medical Officer.

### Systems and structures for implementation of integrated FP/VCT

In this study, majority of VCT health providers (58.8%) are requested by their clients’ to provide information and family planning services. In all the VCT sites studied, at least one type of family planning method was available. Male and female condoms were available in all sites. The facility based sites had all types of family planning methods on site. About half of health providers interviewed do make follow up of referrals of VCT clients largely through use of referral feedback slip (64.7%) and to a lesser extent through physical follow up to the respective health facilities by health providers.

Stakeholders and health providers advocated the need to develop strong monitoring and evaluation systems and building on already existing national tools such as MTUHA in order to harmonize the data tracking and reporting process.

*“Existing FP and VCT data collection tools need to be reviewed to incorporate more entries”* VCT health provider

*“I think we need to review MTUHA to include FP/HIV integrated case e.g. number of FP clients tested for HIV, number of FP clients referred to CTC”* Health care provider.

Qualitative findings showed that some stakeholders see some linkage between FP and VCT in the current set-up. Some participants could point linkages of family planning and HIV testing services at lower levels.

*“Yes, there is linkage between FP and VCT. Health education and counseling sessions and messages links the two services at dispensary the same service provider issue both services”* Health Care Provider.

The main source of funding for counseling and testing as mentioned by various policy makers and service providers were largely from donors including basket fund, government, USAID and CDC. Government, basket fund, USAID and UNFPA were also mentioned as sources of funds for family planning programs. Some of the donors and development partners are currently supporting both family planning and counseling and testing services which create a conducive environment for integration process.

### Health providers and clients perceptions towards FP/VCT integration

**Figure 1:**
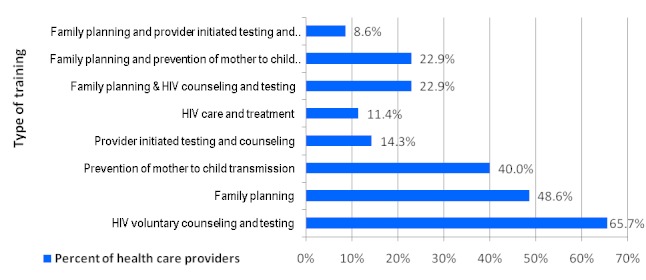
Type of trainings attended by health service providers (N=35)

All health providers interviewed felt that family planning and VCT services could be provided by the same health provider, and about 97% supported FP/VCT integration. The knowledge of health providers on various FP methods varied; with 100% of health providers knowledgeable about male condoms. Vasectomy, bilateral tubal ligation and implants were the most unknown methods of family planning. About 2/3 of the health providers in the surveyed sites (Figure 1) have been trained on counseling and testing services. Slightly less than half are trained on family planning. Less than a quarter of health providers are trained on both FP and VCT services. Almost all health providers (97%) interviewed perceived client workload to be above normal. This finding is supported by focus group discussion where participants felt that health providers will be overloaded and likely to experience burn-out.

*“If there are few care providers they can get burn out resulting into poor service and other client may miss service due to long waiting (time) and decide to go back”* Male service user.

Low staff motivation and lack of training on FP/VCT integration are the main constraints which might hinder implementation of the program as perceived by the health providers. Availability of private and confidential space for providing the services and providers’ attitudes were not perceived as constraints. Although lack of skilled staff and clients’ workload in facilities is major issues, qualitative findings showed that it might be difficult for one service provider to efficient deliver both services.

*“One service provider to perform two activities at the same time is not possible taking into consideration the number of clients”* Female service user.

*“If there are few care providers they can get burn out resulting into poor service and other client may miss service due to long waiting (time) and decide to go back”* Male service user.

Clients’ satisfaction, reduction of waiting time, quality and efficiency of services was perceived to increase if FP and VCT services are integrated.

*“Integration of FP/VCT services will help client to reduce the number of queue she/he is going to make, transport expenses and time”* Female service user.

**Figure 2:**
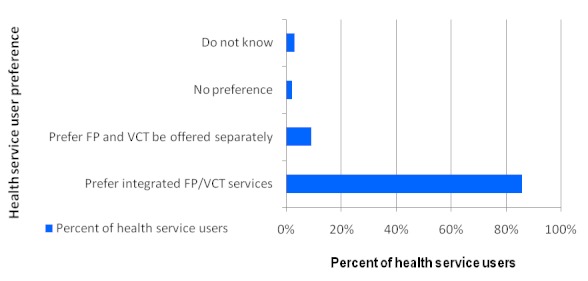
Health service users’ preference regarding service delivery approach for family planning and HIV couseling and testing services (N=35)

**Figure 3:**
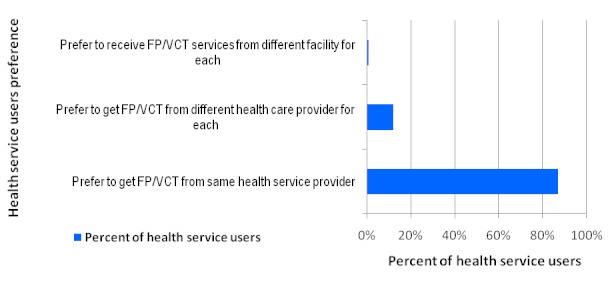
Health service users’ preference regarding provision of family planning and HIV counseling and testing services by same of different providers (N=147)

Majority of services users (86%) prefer to get FP and VCT services within the same site while 87% preferred same provider (Figure 2 and Figure 3). Service users’ focus group discussions supported integration agenda particularly getting the same services in the same room by same provider.

*“The services can be provided in the same building provided there are enough rooms and enough trained staff”* Female service user.

Duo role of condoms in HIV prevention and family planning was commonly pointed out during focus group discussion with service users.

*“HIV infection is transmitted through sexual intercourse and Family planning can be effected by the use of some of the HIV prevention methods like condom”* Male service user.

### Family planning utilization and unmet need among VCT clients

Family planning methods use do not vary much across age groups, however use of family planning methods use increase with age. Majority of females (81.2%) are using at least one family planning method. The use of family planning methods is high among business individuals compared to those employed. No general trend observed in family planning use in relation with education. Majority of married individuals (90%) are using family planning methods and lowest (59%) among singles. The commonest FP methods used are injectables (45.3%) followed by condoms and pills. Female condoms (1.4%) and loops (2.0%) are the least methods used.

Majority of the respondents (91.2%) interviewed were sexually active. Those who ever used FP methods in the past were 69.4%, while current FP users were 74.8%. In the future a large proportion (90%) of respondents said they will use family planning methods. Users commonly reported that all adults of reproductive age need family planning services.

*“VCT client users indicated need for FP services to help mothers and fathers plan how many children they want to have.”* Male service user.

About 60% of females and 40% of males would like to get family planning services at the facilities. Health providers said that, family planning issues were discussed with about two thirds (67.3%) of clients. The use of condoms was discussed both as HIV prevention method as well as family planning method although it is to lesser extent.

The problem of FP services accessibility was perceived to be more prevalent in rural compared to urban settings.

*“In rural setting family planning services are not easily accessed, only a small percentage of general population is using the service”* Male service user.

## Discussion

The policy makers and health care provider in this study supported integration saying that it was feasible only with minor re-arrangement. As found in other studies, lack of clear guidelines for integration and supporting policy is a bottleneck to FP/VCT integration [15]. In this study policy makers and service providers reported few or non-existence of concrete policies on service integration. Findings from this study suggest formulation of comprehensive policies and guidelines that support FP/VCT integration. Policy makers should avoid deficiencies in national plans which might hinder implementation of health interventions [16]. Both quantitative and qualitative findings suggest that service integration will improve access to both the services; however studies have shown little evidence on increased FP services due to suboptimal integration of services [17]. Government and non-government organizations like AMREF which support many VCT sites are a clear opportunity for integration of FP services.

Stakeholders and health providers advocated the need to develop strong monitoring and evaluation systems and build on already existing national reporting tools such as MTUHA in order to harmonize the data tracking and reporting process as has been shown to work in other countries [18]. This study shows that integration of FP and VCT services is particularly crucial at lower level facilities which have few staff. A systematic review by Church indicates that appropriate integrated service delivery model should be based on epidemiological, structural and health systems factors [19]. Although some of the health care providers could provide both VCT and FP services, majority said they would need refresher training on the provision of both services and integration concept to maximize impact and avoid drop in service provision which can result in suboptimal integration [17]. It was clear that majority of donors including Global Fund and USAID are funding VCT and HIV services to larger extent than FP hence integration could be a way forward of tapping these resources as described in a review [19, 20].

In this study, health providers supported integration of family planning into VCT services and felt that, same service providers could offer both services under the same roof. This was contrary to a study done in Mozambique where health providers do not feel that they can influence the behaviors of HIV-positive women effectively [21]. As suggested in another study, change management should feature integration agenda at service delivery level to resolve organizational culture issues and support transformation of implementation [22]. Low staff motivation and limited skills on FP/VCT integration were identified as main constraints for service provision. Availability of private and confidential space for providing the services and providers’ attitudes were not perceived as constraints. This study indicates that majority of service users prefer to get family planning and VCT services under the same roof by the same provider. As in other studies, preference for integration as well as duo role of condoms in HIV prevention and family planning was commonly pointed out by both health providers and service users [12, 21].

Utilization of modern family planning was found to increase with age and majority of females were found to have used at least one method. The commonest family planning method reported in this study was use of injectable contraceptives. A family planning service gap of 60 percent for female and 40 percent for male was found among VCT clients. Integration of family planning into VCT can address this gap and ultimately increase utilization of contraceptive methods particularly duo protection method [18, 23]. In this study, the problem of FP services accessibility was perceived to be more prevalent in rural where both infrastructure and human workforce is limited compared to urban settings. Integration of HIV care, the use of reproductive health services are likely to increase the outcomes of infants born to HIV-infected mothers as indicated in other studies [24].

Facilities and health providers involved were from VCT service delivery points hence the finding may inform integration of family planning in VCT services and not vice versa. Although the time was short, enough and well trained data collectors were used to gather data and required background information within a short time and no service interruption was noted during the study period. Meeting rooms with enough privacy were used to allow adequate discussions during in-depth interviews and focus group discussions. Operational studies involving provision of both services in an integrated manner rather than a cross-sectional study will further inform integration.

## Conclusion

This study suggests that integration of FP and HTC is feasible and acceptable with minor re-arrangement. Joint planning among service providers from both HTC and FP services is a necessary precondition. Involvement of multiple stakeholders is crucial especially at district levels which are critical in enhancing integration. Operational researches on the Quality of services at the integrated settings need to be done together with supportive supervision and refresher trainings to ensure good quality services are being provided.
